# SGCE Myoclonus Dystonia: A Case Report

**DOI:** 10.4314/ejhs.v35i3.11

**Published:** 2025-05

**Authors:** Endayen Deginet, Melatemariam Tewoflos, Yared Nigusie Abebe

**Affiliations:** 1 Department of pediatrics and child health, school of medicine, College of health and medical sciences Saint Paul's Hospital Millennium Medical College, Addis Ababa Ethiopia; 2 Department of Neurosurgery Saint Peter's Specialized Hospital, Addis Ababa Ethiopia

**Keywords:** SGCE gene, Myoclonus Dystonia, Child, Case report, Ethiopia

## Abstract

**Background:**

SGCE myoclonus dystonia is a rare genetic movement disorder caused by mutations in the SGCE gene. It typically presents in childhood and is characterized by myoclonus and dystonia. The inheritance pattern is autosomal dominant with maternal imprinting. Patients often exhibit associated psychiatric disorders, although cognitive function remains intact. Treatment includes antiseizure medications for myoclonus and anticholinergic agents or botulinum toxin for dystonia. Deep brain stimulation may be used for severe cases.

**Case Presentation:**

We present the case of a 2-year-old female who developed dystonia in her lower limbs over eight months, followed by myoclonus affecting her trunk and extremities. She met her developmental milestones and had no family history of similar conditions. Genetic testing revealed a pathogenic variant of the SGCE gene. She showed improvement with clonazepam.

**Conclusion:**

Myoclonus dystonia should be considered in patients presenting with early-onset myoclonus and dystonia. Genetic testing can confirm the diagnosis by identifying SGCE gene mutations. We hope this case increases awareness of SGCE myoclonus dystonia and encourages further research.

## Introduction

SGCE myoclonus dystonia, also known as DYT11, is a rare movement disorder characterized by myoclonus (jerky movements) and dystonia (muscle contractions leading to twisting and abnormal postures). It is caused by autosomal dominant pathogenic variants in the epsilon-sarcoglycan (SGCE) gene (1,2,3). The SGCE gene encodes a ubiquitous membrane protein that is part of the dystrophin-associated glycoprotein complex found in the brain. Over 100 pathogenic variants of the MYC/DYT-SGCE gene, located on chromosome 7q21, have been identified. Due to maternal allele silencing, less than 5% of patients with myoclonus dystonia exhibit a maternally inherited mutation, with most cases displaying paternal inheritance or arising from de novo mutations (1).

The epidemiology of myoclonus dystonia is not well-established, but some studies estimate its prevalence to be 1 in 500,000 in Europe. It typically presents in childhood, with an average age of onset of 10 years. Symptoms tend to develop earlier in girls than in boys (2). Myoclonus dystonia generally presents in the first decade of life or adolescence. The primary symptoms are upper extremity dystonia (often described as “writer's cramp”) and cervical dystonia, followed by myoclonus of the upper body. Stress or exercise can exacerbate symptoms. While myoclonus dystonia predominantly affects the upper body, involvement of the lower limbs is less common in patients with SGCE myoclonus dystonia. Psychiatric comorbidities, including obsessive-compulsive disorder (OCD), anxiety, and alcohol use disorder, are frequently observed (1,3,7).

The course of the disease is often benign, with the potential for spontaneous remission. However, in certain cases, the condition may persist into adulthood, spreading to previously unaffected areas or increasing in severity (5). Diagnostic criteria exist for adults but are not applicable to pediatric populations, as they include factors such as motor sensitivity to alcohol and the co-occurrence of psychiatric symptoms, which are not typically observed in early childhood (6).

Symptomatic management primarily involves antiseizure medications for myoclonus and therapies for focal dystonia. Common first-line treatments for myoclonus include benzodiazepines or valproate. For dystonia, anticholinergic medications and botulinum toxin injections are frequently used. In cases with more widespread symptoms, carbidopa-levodopa or sodium oxybate may be considered. More recently, deep brain stimulation (DBS) targeting the globus pallidus internus or the ventral intermediate nucleus of the thalamus has been reported for severe disease management (5,6).

We report the case of a 2-year-old female from Addis Ababa, Ethiopia, who presented with dystonia and myoclonus starting at the age of 1 year and 4 months. Her genetic test was positive for a pathogenic variant. The patient's mother provided informed consent for the publication of this report. We hope this case contributes to clinicians in Ethiopia better understanding and diagnosing early-onset primary dystonias in children.

## Case Presentation

A 2-year-old female, the first child of non-consanguineous parents, presented with an eight-month history of dystonic posturing in her lower limbs. Initially, the dystonia was intermittent, affecting the lower limbs for 1–2 minutes, occurring 6–7 times per day. A few weeks later, she developed brief myoclonic jerks involving the trunk and limbs, occurring multiple times daily. There was no family history of similar illness. Behavioral changes, including excessive crying, irritability, and aggressive behavior, were noted after the onset of symptoms.

She was born after an uncomplicated pregnancy and delivery, with normal growth and developmental milestones during her neonatal period.

Physical examination revealed normal vital signs and anthropometric measurements, with no dysmorphic features. Neurologically, she was conscious, with normal pupil reactions and eye movements. Limb power was 5/5, with normal deep tendon reflexes and no sensory loss. Laboratory blood work was normal.

Multiplex Ligation-dependent Probe Amplification (MLPA) was performed and showed duplication in exons 3 and 4 of the SGCE gene on 7q21.3. The patient's brain MRI was normal. She was started on clonazepam 0.5 mg daily, resulting in a marked reduction in dystonia frequency. Parents were counseled on the condition and the importance of regular neurological evaluations.

## Discussion

Our patient presented at a young age, which is consistent with the typical onset of myoclonus dystonia, usually occurring in the first or second decade of life (4). She initially exhibited lower limb dystonia, followed by myoclonus. While myoclonus dystonia typically affects the upper body, involvement of the lower limbs has been reported in about 25% of cases, more commonly in girls. Lower limb dystonia is seen in over half of the cases when the condition presents in the first decade of life. This involvement is less common when the disease presents later in life (3,4,6).

Psychiatric conditions such as anxiety, depression, panic attacks, obsessive-compulsive disorders, and personality disorders are commonly seen in patients with myoclonus dystonia. In our patient, excessive crying, irritability, and aggressive behaviors were noted; however, it is too early to diagnose psychiatric illness as she is only 2 years old (4). Children diagnosed with myoclonus dystonia at an early age may experience symptom progression, with previously unaffected body parts becoming involved over time. These patients require close medical follow-up to monitor for the development of new behavioral abnormalities as they progress through childhood and adolescence (3). Cognitive impairment is generally not observed in these patients, and our patient has met all developmental milestones (4).

In our patient, MLPA confirmed a duplication in exons 3 and 4 of the SGCE gene on 7q21.3. Myoclonus dystonia follows autosomal dominant inheritance, and the majority of affected individuals display paternal inheritance due to maternal allele silencing. Less than 5% of patients with myoclonus dystonia express a maternally inherited mutation, and the remaining individuals may have de novo mutations (2,5). Our patient has no family history of similar illness, suggesting a de novo mutation.

Certain antiseizure medications, such as valproate, levetiracetam, zonisamide, and benzodiazepines like clonazepam, as well as topiramate, have been found to improve myoclonus in patients with myoclonus dystonia. The dystonia component of the condition may be alleviated through the use of anticholinergic medications and botulinum toxin injections (5). Our patient showed improvement after starting clonazepam.

Studies have shown that deep brain stimulation (DBS) is an effective therapy for SGCE myoclonus dystonia (6); however, the lack of DBS facilities in resource-limited settings, such as Ethiopia, poses a challenge for its application.

In conclusion, SGCE myoclonus dystonia is a rare movement disorder characterized by a combination of dystonia and myoclonus. The disorder is caused by mutations in the SGCE gene, which follows an autosomal dominant inheritance pattern with maternal imprinting.

Our patient presented with dystonia initially affecting the lower limbs, followed by myoclonus affecting the trunk and extremities. She has met her developmental milestones and has no family history of similar illness. Her genetic test revealed a pathogenic variant of the SGCE gene, confirming the diagnosis of SGCE myoclonus dystonia. She showed improvement after starting clonazepam.

We hope this case will raise awareness among clinicians in Ethiopia about SGCE myoclonus dystonia and encourage vigilance in diagnosing this condition. It may also serve as a foundation for further study.

We recommend that clinicians consider primary dystonia in children presenting with early-onset movement disorders, especially when brain imaging is normal. Genetic testing should be part of the diagnostic workup to ensure early diagnosis
